# Protection Motivation Status and Factors Influencing Risk Reduction Measures among the Flood-Prone Households in Bangladesh

**DOI:** 10.3390/ijerph191811372

**Published:** 2022-09-09

**Authors:** Md. Sazzad Ansari, Jeroen Warner, Vibhas Sukhwani, Rajib Shaw

**Affiliations:** 1 Disaster Risk Management Professional, Bhaluka 2240, Bangladesh; 2 Social Sciences Group, Wageningen University, P.O. Box 8130, 6700 EW Wageningen, The Netherlands; 3 Graduate School of Media and Governance, Keio University, 5322 Endo, Fujisawa 252-0882, Japan

**Keywords:** flood risk, threat appraisal, coping capacity, protection motivation, Bangladesh

## Abstract

Bangladesh, a low-lying deltaic country, experiences recurrent floods. To reduce the subsequent losses and damages, self-preparedness measures are imperative. In that context, the present study attempted to assess the flood protection motivation status of local flood-prone households through the evaluation of threat and coping capacities, as well as the identification of the factors that influence preparedness actions. Using Protection Motivation Theory (PMT), this study employed a mixed-method approach at three different flood-prone locations in Bangladesh: (1) Type 1 settlement, an area with ‘hard (flood embankment)’ flood risk reduction measures; (2) Type 2 settlement, without any risk reduction measure; (3) Type 3 settlement, with ‘soft’ measures put in place as part of NGO-led disaster risk reduction interventions. The study findings revealed a comparatively higher protection motivation status among the respondents living in the Type 3 settlement, in terms of evaluating the flood risk and capacity to take flood preparedness measures. The correlation analysis further illustrated that the factors of the perceived probability and severity of flooding, coping capacity, previous flood experience, reliance on NGO interventions, and gender status had an influence on the protection motivation of flood-prone households. Remarkably, no such influence was found for socio-economic factors such as education and income. It is hoped that the study findings can support the future decision-making process for designing preparedness interventions for communities in flood-prone areas.

## 1. Introduction

Floods are widely recognized to be among the most frequent and widespread disastrous types of events, causing substantial damage to human societies [1,2] and posing a threat to sustainable development [3]. Other factors, such as population growth, rapid urbanization, and climate change, have further exacerbated the implications of flooding over the years, particularly in coastal areas characterized by high-density population and economic assets [4]. Even as adaptation measures are progressively being put in place, global flood-related losses are projected to rise in the coming years due to the increase in flood probability, subsidence, and sea-level rise [1,5]. 

Bangladesh, a low-lying, densely populated country, consists of the floodplains of the Ganges, Brahmaputra, Meghna, and several other rivers [6]. Correspondingly, it is also recognized as one of the most flood-prone countries worldwide [7,8,9]. Therein, the restless rivers, mostly of foreign origin, continue to raise serious concerns about riverine floods, while Bangladesh has limited control over the upstream water source areas. Resultantly, the country is faced with the serious destruction of properties and danger to the lives and livelihoods of people [10,11]. 

In 2017, the country experienced severe floods causing the inundation of a huge landmass, the destruction of houses, the displacement of people, and the damage to croplands in the northern parts of the country [12]. However, studies have suggested that people living in the flood-prone areas are partly aware of survival strategies, including building platforms out of reeds, banana shoots for animals, fixing their wooden bed just below the roof and cooking on potable ovens made during the winter season [13,14]. This component is important, as the precautionary measures adopted by flood-prone households hold the key to reduce loss and damages [15,16], and evidence has suggested that 80% in monetary value can be saved by taking protection measures in the urban setting [17]. The scientific literature has demonstrated that people prone to coastal and riverine floods take anticipatory precautionary measures, including the dissemination of early warning, [18,19,20], storing emergency equipment at home [21,22], raising awareness about damage insurance [23], making furniture flood proof, and raising protection barriers to prevent water from entering the house [24,25,26]. Hence, people living in flood-prone areas eventually adopt mitigation measures by themselves to complement existing public flood risk reduction measures [12,27]. However, the level of preparedness action taken varies with the capacities, and in many cases, the households’ capacity to facilitate flood protection is inadequate for many reasons that need to be further explored [28].

Thus far, the studies conducted in Bangladesh have mostly focused on the human impact of riverine floods and the adaptation and mitigation measures associated with traditional knowledge to survive the crisis [6,7,10,29,30,31,32,33]. In recent years, a few scientific studies have also been conducted on household-level flood mitigation measures [34], the examination of the adaptation strategies of riverine communities [35,36], motivation towards taking action [12], and household responses to the flood in 2017 [37]. However, apart from a recent study conducted by Mondal, et al. [12], inadequate attention has been paid to assessing the determinants and factors that motivate people to take flood preparedness measures using Protection Motivation Theory (PMT), as proposed by Rogers [38], in the context of developing countries. Against that backdrop, this paper aimed to investigate the protection motivation status of flood-prone households living on the banks of the Jamuna river using the PMT lens. The present research study was conducted in three specific and reasonably proximate settlements associated with various structural and non-structural flood risk reduction measures to derive cross-case lessons on the protection motivation status and the factors influencing the risk reduction behaviors of the households living in those areas.

Broadly, this manuscript comprises five sections, including the Introduction (Section 1). Section 2 provides a precise description of the adopted research methods, after establishing the conceptual framework and introducing the case study area. Section 3 presents the study results, which are then followed by a wider discussion in Section 4. Lastly, Section 5 summarizes the key conclusions and research limitations.

## 2. Materials and Method

### 2.1. Conceptual Framework

Protection Motivation Theory (PMT), adopted for this study, was previously used by Grothmann and Reusswig [17] in the context of Germany. In that study, the theory was adapted from Rogers, who proposed it in 1975 [38]. Based on the overall understanding, PMT proposes three key components. First, threat appraisal (or risk perception) describes how a person assesses threat probability and damage potential to valuable things, assuming no changes in his or her own behavior. Here, risk perception can be defined as people’s subjective judgment anticipating an uncertain event and associated consequences, especially where something of human value is at stake [39,40]. This uncertainty evolved from risk perception plays an influential role in attributing human behavior in uncertain situations [41]. Further, ‘Threat Appraisal’ comprises three subcomponents: (1) perceived probability, a person’s expectation of being exposed to the threat, such as a flood reaching their house; (2) perceived severity, a person’s estimate of how harmful the consequences of the threat would be to the things that they value if the threat were to actually occur (e.g., the belief that a flood in the area would harm valued things, such as home or property); (3) fear plays an indirect role in threat appraisal by affecting the estimate of the severity of danger.

The second component of PMT is coping appraisal, whereby a person evaluates their ability to cope with and avert being harmed by the threat, along with the costs of coping. This component takes place after the threat appraisal process, and only starts if a specific threshold of threat appraisal is passed. It has three subcomponents: (1) perceived protective-response efficacy, the belief that protective actions are in fact effective in protecting oneself or others from being harmed by the threat; (2) perceived self-efficacy, the person’s perceived ability to perform or carry out these protective responses; (3) perceived protective-response costs, the assumed cost of performing a preventive response, including not only monetary cost but also the factors of time and effort.

In PMT, protective responses are those that seek to prevent monetary or physical damage if an event occurs and are resorted to if threat appraisal and coping appraisal are high. On the contrary, non-protective responses—including the denial of the threat, wishful thinking, and fatalism—do not prevent monetary or physical damage but only the negative emotional consequences of the perceived risk, such as fear. A person adopts non-protective responses if their threat appraisal is high but coping appraisal is low. If a person chooses a protective response, they first form a decision or intention to act, labeled ‘protection motivation’, which does not necessarily lead to actual behavior due to actual barriers, such as a lack of resources, i.e., time, money, knowledge, or social support. Actual barriers, according to the theory, are circumstances that act as barriers to achieving a protective-response goal. These barriers, unforeseen in the motivational stage of a protection response, can be aspects such as costs, knowledge, and physical capabilities. 

Apart from the two main components of PMT, a few other aspects have been added to the model from the perspective of flood damage prevention. One is ‘Threat Experience Appraisal’, which assesses the severity of a threat experienced by people in the past; it should help people to assess future threats and motivate them to take precautionary measures. Secondly, the ‘Reliance’ of people on public protection interventions (e.g., flood embankment) can influence the preparedness status. Household-level damage prevention can be redundant if public agencies successfully build levies to prevent floodwaters from reaching people’s doorsteps, likely because residents at risk may take less precautionary action themselves if they rely on the efficacy of public or administrative flood protection. 

Based on that understanding, this study intended to examine the relations among the variables of threat appraisal, threat experience appraisal, coping appraisal, reliance (flood embankment and DRR interventions), socio-economy (gender, education, and monthly income), and risk exposure (distance of household from river and embankment) to explore the influential factors that contribute to the protection motivation of households. For the better comprehension of readers, a precise set of indicators of the adopted PMT components in the context of the present research study is provided in Table 1.

### 2.2. Study Area

**Figure 1:**
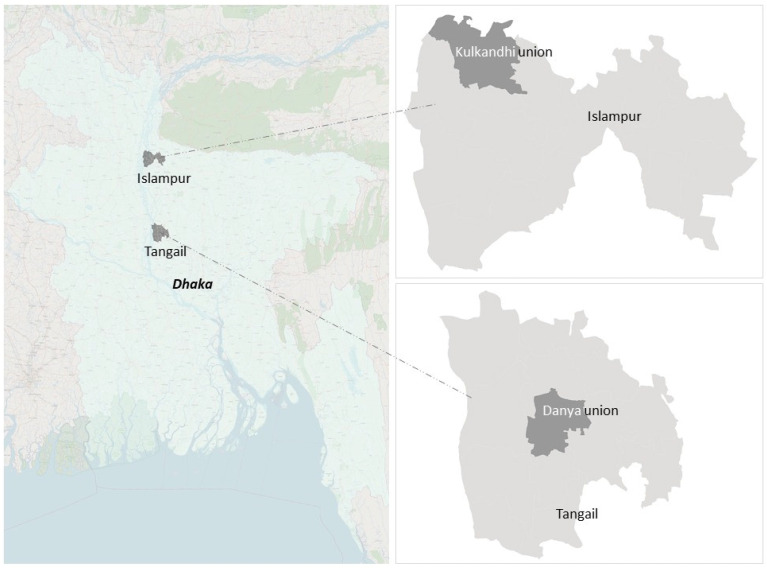
Location map of the study areas: Danya Union in Tangail Sadar Upazila, Tangail District, and Kulkandi Union in Islampur Upazila, Jamalpu District (background image source: Openstreet). Union, Upazila, and District represent, respectively, the fourth, third, and second administrative layers of the government of Bangladesh.

Three specific settlements (in this study, we mean a village/place where people live) from Bangladesh were selected for this study. Two of these villages are situated in Tangail Sadar Upazila in Tangail District (location shown in Figure 1); the third settlement is situated in Islampur Upazila in Jamalpur District (location shown in Figure 1). These specific study locations were identified based on three specific criteria: (1) identifying settlements vulnerable to floods, (2) identifying diverse settlements located by the bank of/nearby a major river or its tributaries, and (3) identifying settlements where different types of structural and non-structural flood risk reduction measures are implemented. The precise characteristics of each of the three selected settlements are described in the following subsections. 

#### 2.2.1. Type 1 Settlement: Fatepur—Area with Structural DRR/Flood Protection Embankment

Fatepur is situated by the Jamuna river in Tangail District. Until l995, it experienced floods every year during the monsoon season. Thereafter, an earthen flood protection embankment was constructed under Compartmentalization Pilot Project (CPP), known as Flood Action Plan 20 (FAP-20) during 1990–1995. The objective of this structural intervention was to secure infrastructural development against the impact of floods and to intensify the agricultural production in the region, to comply with the food security agenda of the Bangladeshi government [42]. Since 1995, the local inhabitants have not experienced flooding apart from a few small-scale embankment breaches in 2004, 2016, and 2017. These breaches caused waterlogging behind the embankment, due to which people suffered from damage to households, agricultural crops including vegetable gardens, and the inundation of fishery resources. While a few micro-credit organizations have implemented credit projects here, no other specific DRR interventions are reported in this area apart from the embankment. 

#### 2.2.2. Type 2 Settlement: Char Fatepur—Area without any DRR Measure from External Stakeholders

Char Fatepur, also situated in Tangail District, is located away from the flood protection embankment and is exposed to the flooding of the Jamuna river. During the monsoon season, this area usually becomes isolated from the mainland, and boats are the only way to reach the village. People in this area experience monsoon floods on varied scales on a regular basis. In 2017, local people suffered from the loss of agricultural crops and domestic animals; damage to their house facilities, tube wells, toilets, and mud-made cooking stoves; and waterborne diseases. Herein, apart from receiving emergency relief support, no specific flood risk reduction interventions by the government nor nongovernmental organizations have been reported; however, local people do repair houses by themselves sometimes, not necessarily considering future flood risk. There are a few micro-credit organizations through which people have access to loans when needed.

#### 2.2.3. Type 3 Settlement: Kulkandi—Area with Non-Structural and Minimal Structural Measures

Kulkandi is located besides the Jamuna river in Islampur Upazila, in Jamalpur District in the north-central part of Bangladesh. The inhabitants of this area have experienced floods of varying intensity on different occasions, specifically in 2017. People suffered from the inundation of agricultural crops; partial damage to houses, toilets, and tube wells; and water-borne diseases, with children being affected the most. Here, various DRR interventions have been implemented by several national and international NGOs. The major activities implemented under these projects include flood awareness campaigns, the development of local institutions and disaster management plans; the formation and strengthening of local disaster management committees; livelihood support; and cash for work activities for raising the plinths of houses, toilets, and tube wells and the increase in the road height where necessary using mud. In NGO interventions, raising the plinth is considered as a minimal structural measure for household flood protection. Furthermore, the presence of credit organizations is also reported in this area.

### 2.3. Data Collection Methods

Building on the PMT components, a mixed-method approach was adopted for the study, mainly utilizing quantitative methods to collect the relevant data. The time period in consideration for this study was from November 2017 until the end of 2018. The detailed description of the adopted research methodology is explained in the following sub-sections.

#### 2.3.1. Surveys

A household survey was conducted with the representatives of 90 families (30 houses each in the 3 selected study locations), comprising of 58 male and 32 female participants. Conducted with the assistance of trained volunteers, each interview took around 35 min. A systematic random sampling procedure was employed to select families in the three villages. To collect a representative sample from the entire community, the center of the village was first defined with the help of a local elderly person. Thereafter, the surveys were initiated from the village center in all four directions (north, south, east, and west) to reach out to the families. A semi-structured questionnaire was developed for the survey, wherein the key questions were in line with PMT components, including threat appraisal, coping capacity, and precautionary and non-precautionary measures, specifically addressing the indicators defined in Table 1. The questionnaire was pre-tested in the field to check whether the questions were contextualized enough to obtain the required data from the respondents and was accordingly improved (please see Appendix A). 

The age range of the survey respondents was between 26 and 65 years. More than half of respondents living in the Type 1 (57%) and Type 2 (67%) settlements and less than half (37%) of respondents from the Type 3 settlement were found to be illiterate, as they had not completed primary school (i.e., classes one to five). The average monthly family income was found to be 32.86 USD (1 USD = 84 Bangladeshi Taka) in the Type 1 and Type 2 settlements and 65 USD for the families from the Type 3 settlement. The primary livelihood was agriculture, and the rest depended on rickshaw pulling, fishing, and working with NGOs. Only two respondents living in the Type 3 settlement were reported to have a secondary source of income. 

#### 2.3.2. Focus-Group Discussion (FGD)

The quantitative findings derived through the household survey were complemented by six FGDs (two in each off the three selected study locations), which were conducted with local community members in 2018. Therein, an average of 8 participants attended each session for 45–60 min, comprising a mixed gender representation. Although occupational diversity was reported among the FGD participants in all three locations, a list of standard questions, aligned with Table 1, related to PMT components were asked during all the FGDs to keep the findings harmonized as well as to facilitate a comparative analysis. Other key areas of discussion included flood risk, previous flood experience and associated damage scenarios, coping strategies and motivation for flood preparedness, reason for the demotivation of people, and effects of types of assistance received from different stakeholders on households. Emphasis was put on realizing free-flow and interactive discussions, wherein the participants could also raise counter questions and debate amongst themselves. To ensure the clarity of the statements and explanations, the FGDs were moderated with probing/follow-up questions.

### 2.4. Recording, Organizing, and Analyzing the Data

Statistical Produce and Service Solution (SPSS), Version 12, was employed to analyze the data and for the visualization of the data related to indicators mentioned under each of the PMT components. There were only a few missing responses; for instance, a few respondents did not want to declare their monthly income. Additionally, the SPSS data set was cross-checked with the hard copy of the filled-up question papers to avoid errors made during data entry. Apart from a simple frequency analysis and the preparation of graphs, a correlation analysis was performed to further explore the relationships among the variables related to the PMT components and the flood protection actions found in the relevant literature [17,18,38], as mentioned in Table 1. The Spearman correlation was considered due to the asymmetrical distribution of the variables, as the values occurred at irregular frequencies, and the mean, median, and mode occurred at different points. To amplify the research findings, the quantitative data from the surveys were first integrated with qualitative explanations related to the protection motivation status of the respondents later gathered from the FGDs. For instance, the quantitative status of the motivation for taking preparedness action was combined with the qualitative discussion on the motivation of respondents. 

## 3. Results

### 3.1. Threat Appraisal

#### 3.1.1. Intensity and Severity of Future Flooding

The study findings revealed that the majority of respondents living in the Type 2 and Type 3 settlements and half of respondents from the Type 1 settlement foresaw severe flooding happening in the coming years. Half of respondents from the Type 1 settlement (living by the flood protection embankment) could not provide a response and were not sure about the future. Further, the respondents were asked to mark the intensity of future floods, along with the severity of the damage caused by the event using a five-point Likert scale denoting very high to very low (as in Table 2).

In the Type 1 settlement, among those who responded, 53% assessed future flood intensity to be ‘low to very low’. The participants in the FGDs also shared that they did not experience any flood event compared with what happened away from the flood protection embankment, and they considered a flood less likely to happen, even though a few of them mentioned experiencing small-scale embankment breaches and waterlogging situations in the past. Additionally, around 47% of respondents replied with a medium to high possibility of flooding and damage caused by the event, where they highlighted several breaches of the embankment that had happened in previous years due to the inadequate maintenance of the flood protection embankment by the designated authorities, including the Bangladesh Water Development Board (BWDB) and the local Water Management Committee. 

In contrast, the majority of respondents (77% on average) living in both the Type 2 and Type 3 settlement areas marked the intensity of the flood disaster and severity of the damage as high to very high. The FGDs in these areas suggested that the flood risk perception was linked to recent and previous flood events that community people had experienced in 2017 and earlier. They reported that the flood water level during the 2017 floods was higher, along with the damage to households structures, as compared with the previous years. 

#### 3.1.2. Fear of Future Floods

As observed in Table 2, the level of fear of future flood disasters among the respondents with soft measures in the Type 3 settlement was greater than that in the other areas. Around 70% of respondents living in the Type 3 settlement mentioned a ‘very high’ level of fear of future floods and that they were scared of losing their valuable assets and lives. The FGDs in all three settlement types found that the water height and velocity during the flood of 2017 were greater than those in previous years.

The majority of respondents (47%) living with hard measures in the Type 1 and Type 2 (37%) settlements saw their fear as high. Further, the people who lived by the embankment (Type 1) had witnessed several breaches in various points of the flood protection embankment, and this was a likely reason why people were scared about bigger breaches of the embankment and were expecting heavy losses of lives and assets in the area. Another potential reason could be the severe waterlogging situation that happened in 2017 and damaged agricultural crops and assets.

The FGDs conducted in the Type 2 settlement revealed that the area is detached from the mainland by the tributary of the Jamuna river and that if something were to happen, they would not be able to quickly move to other locations, unless they had a plan and associated arrangements, including a boat to move out during the flooding. However, 24% of survey respondents also mentioned low levels of fear, as the flood is a regular event, and they had been facing this since childhood. 

#### 3.1.3. Evaluation of Damage Caused by Hypothetical Flooding in the Future

The study findings implied that the damage perception of respondents due to future flooding was associated with factors such as agricultural-crop loss, the loss of domestic animals, family health, and fishery business in all three settlements. The majority of respondents in all three areas highlighted partial to total damage to the households due to future flood events. In the FGDs, the respondents living in the Type 2 and Type 3 settlements shared that the severity of floods had gradually increased over time. People from the Type 1 settlement also feared the possibility of experiencing severe waterlogging due to breaches of the flood protection embankment in the future, which might cause partial damage to the plinths/basement of houses and toilets, as these are made of mud and tin. 

**Figure 2:**
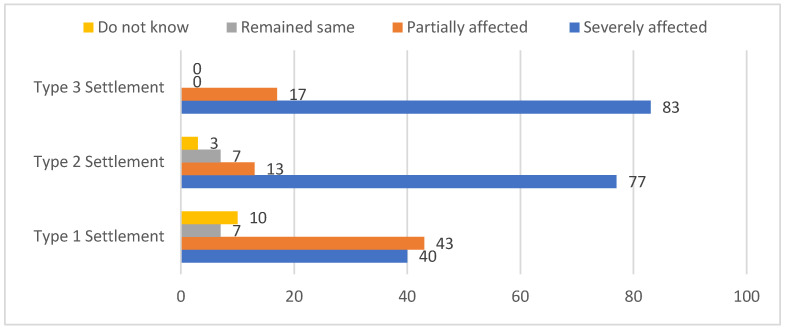
Perception (in %) of respondents (n = 90) on the overall impact of a hypothetical flood on their livelihood in three settlements through a four-point Likert scale ranging from ‘Severely affected’ to ‘Do not know’. Source: survey.

For agricultural crops, on average, more than 70% of respondents in the three areas estimated severe damage to agricultural crops due to future flooding. They mentioned the inundation of agricultural seed beds and losing harvested Jute crops in the flood event in 2017. On average, 30% of respondents from the Type 2 and Type 3 areas perceived that a few chickens and ducks might die during a flood. Regarding health, the majority of respondents living in the Type 2 (95%) and Type 3 (75%) areas reported a possibility of family members suffering from waterborne diseases during and after a flood event. As for fishery business, only 10% of respondents from the Type 1 settlement mentioned the damage to fishery due to the inundation of cultivated ponds with high water levels during flooding/waterlogging, as fish would escape as the ponds would become open-water resources. Fish cultivation in ponds was not common in the other two areas, i.e., Type 2 and Type 3 areas; thus, the respondents could not predict future fishery losses. The majority of respondents living in the Type 3 (85%) and Type 2 settlements (80%) and more than one-third of respondents from the Type 1 settlement reported that their daily life would be severely affected if a flood were to happen in the future (also shown in Figure 2).

### 3.2. Coping Appraisal: Preparedness for Hypothetical Flooding

**Figure 3:**
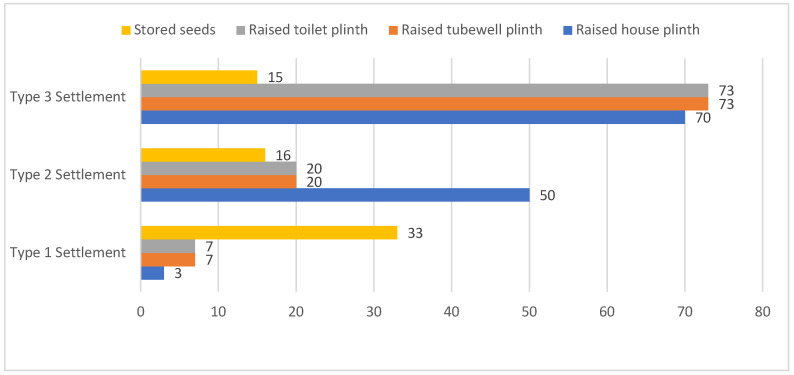
Status (%) of structural and non-structural preparedness measures taken by respondent in all three settlements (n = 90). Source: survey.

**Figure 4:**
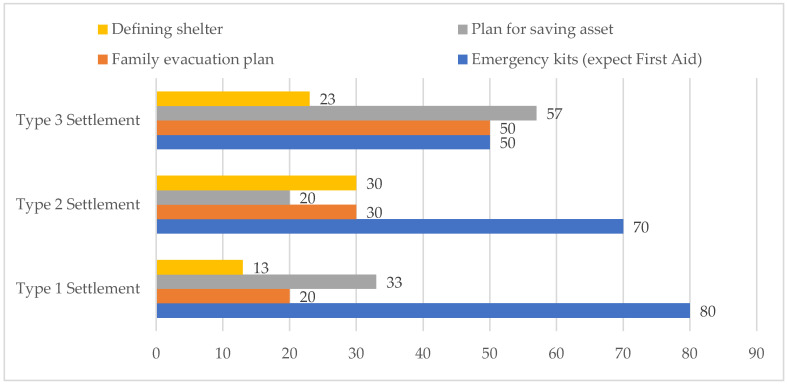
Status of family-level preparedness shared by respondents (%) in three settlements. The study entailed 90 respondents in three areas with equal sample size (n = 30). Source: survey.

More than 80% of people living in the Type 3 settlement, 65% of respondents from the Type 2 settlement, and less than half of respondents living in the Type 1 settlement were reported to have made preparations for future flood disasters. The status of individual preparedness measures is summarized as follows:

- Two-thirds of respondents from the Type 2 and Type 3 areas were found to receive early flood warning/preparedness messages via electronic media, including radio and television, and through the Community Disaster Management Committee (CDMC) formed by NGOs. In the FGDs, community people underlined that they had connections with CDMC members and BDRCS volunteers and staff, who informed them if there was a flood warning in the monsoon season;
- On average, 74% respondents in all settlements mentioned having dry food, including puffed rice, molasses, flattened rice, and biscuits at home, which could be used during emergencies. The qualitative findings suggested that dry food was not primarily stocked in view of a flood situation in any of the areas; rather, respondents living in the Type 1 settlement reported that they kept these food items to make the children happy and to sometimes entertain guests;
- On average, 72% of respondents living with soft measures in the Type 3 area and 50% of Type 2 settlement respondents had raised the plinth of their houses to avoid having flood water entering their houses (refer to Figure 3). In the FGDs, participants from the Type 3 settlement shared that they had received cash support from the Bangladesh Red Crescent Society (BDRCS) and other agencies for strengthening the structural measures to reduce the damage caused by flooding. In the Type 2 area, the physical observation of the houses during the interviews and transect walk in the village further revealed that almost all the houses were built on elevated areas with respect to the normal village road. However, half of respondents did not raise their basements. People living by the flood protection embankment in the Type 1 settlement were found not to have adopted structural measures, as only an average of 6% of respondents replied ‘yes’ to the question about having raised the plinths of the house, toilet, and tube well. More than two-thirds of respondents reported that they had not faced any flood in the last 20 years due to having the flood protection embankment built by the Government of Bangladesh;
- One-third of respondents living in the Type 1 area and, on average, 15% of the respondents from both the areas with soft and no DRR measures stated that they stored some seeds to use after flood events or any other situation (as shown in Figure 4);
- On average, 65% of respondents from both Type 1 and Type 3 settlements and 87% respondents living in the Type 2 settlement did not have any savings in the bank or any other institution. The FGD participants in all three areas uncovered that their monthly income was very low, due to which they were unable to save money for any unforeseen crises in the future;
- Regarding the availability of emergency kits, which include a radio (to listen to news updates), a torch light/candle, fire matches (to make fire immediately after the flood), and a first-aid-kit box, the majority of respondents in all settlements confirmed having all the above-mentioned items, except for a first-aid kit. More than two-thirds of respondents living in the Type 1 settlement agreed with the importance of having an emergency kit at home not only in case of flood events but also as regular equipment in the house, except for the first-aid box, which is expensive and critical to maintain;
- Around 50% respondents from the Type 2 area and 30% of those living in the Type 3 settlement confirmed having an informal evacuation plan at the household level. The FGD participants in these areas reported that they held conversations with neighbors about what to do and how to save the whole family including children during flooding. Such discussions included managing boats to relocate the family members to other places. In the Type 2 and Type 3 settlements, the FGD participants mentioned that households having boats often support others by accommodating family members for relocation during crises. Additionally, one-fifth of respondents living in the Type 1 area said they were thinking about relocating the family to other places, considering severe waterlogging events in the future;
- In all three selected settlements, respondents were found to be concerned about saving valuable assets. More than half of respondents living in the Type 3 area and one-third of Type 1 settlement respondents mentioned that they thought about saving domestic animals, ornaments made with gold (mostly for females), important papers on assets, etc.;
- Respondents living in the Type 3 settlement stated that due to the lack of any flood protection embankment, they would need to take shelter on high-elevation roads along with their families and domestic animals. Regarding the temporary relocation of affected families, one-third of respondents living in Type 2 Settlement and one-fifth of Type 3 settlement respondents said they would take shelter on high roads;
- Lastly, people living with hard measures in the Type 1 and in Type 2 settlements reported that they did not have a community plan to collectively manage flooding. More than 50% of respondents from the Type 3 area said they had a community disaster management plan, which was developed by Community Disaster Management Committee (CDMC) members along with community people with technical support from BDRCS project staff. In discussing the effectiveness of that plan in the 2017 flood, it was noted that members of the committee had conducted a small-scale search and rescue operation to help people.

### 3.3. Non-Protective Response

More than half of respondents (57%) living by the flood protection embankment (Type 1) and one-third of those (35%) living in the Type 2 settlement reported that they were not motivated or willing to prepare for floods (refer to Table 3). The majority of respondents with non-protective responses in all three settlements recognized the flood as a natural event, so they argued that it would be best not to do anything, as it would seem an act against mother nature, over whom they do not have any sort of control. One-third of respondents living in the Type 1 area were reported not to be motivated for flood preparedness, as they mentioned that the flood would not harm them. The survey also found the dependency of people living in the area with soft risk reduction measures on others, which included external stakeholders or even the neighbors. Many NGOs have worked in this community with disaster risk reduction and resilience projects, and these people have always received some assistance from them. This could likely be a reason why the respondents were quite sure that someone would come and save them. 

### 3.4. Explanatory Factors for Flood Protection Motivation

#### 3.4.1. Reliance on Protection Measures

According to PMT, the reliance of people on flood protective infrastructures or DRR interventions could have an impact on individual- or family-level preparedness for flood disaster. Through this study, it was found that on average, more than two-thirds of respondents living in the Type 1 and Type 2 settlements relied on soft DRR measures, which are implemented by NGOs and governments. Additionally, half of respondents living in the Type 3 settlement, which included DRR/resilience building interventions implemented by NGOs, shared that these projects could help to make them prepared and thus could save lives and assets from flood disasters. In the Type 3 settlement, the FGDs also found that soft DRR measures provided information and knowledge on flood disasters and livelihood assistance, so that affected people can have resources to cope with the crisis. 

#### 3.4.2. Threat Experience/Appraisal

The majority of respondents living in the Type 1 settlement reported that due to embankment breaches, flood water had been entering their house yard, inundating fishponds, agricultural-crop fields, and vegetable gardens. In both Type 1 and 2 settlements, participants in the FGDs also mentioned losing domestic animals and damage to mud-made cooking stoves, sanitary latrines, and managing fodders. It was stated that the tube wells were in good order, as flood water did not enter them. A few respondents mentioned health challenges such as cold, fever in children, and adults suffering from itching in the legs and hands, as well as fever. In the Type 1 area, FGD participants reported that a total of 15–20 households were inundated, affecting 200–250 families. 

In the Type 3 settlement, farmers who preserved their jute crop were washed away by the sudden high-speed water flood in 2017. A total of 8–10 ponds of varying sizes were inundated during the flood in the Kulkandi village. Flood water caused partial damage to the households due to the washing away of the mud of basement of houses and toilets. 

### 3.5. Correlation Analysis

The analysis showed that ‘Threat Appraisal’ was correlated with ‘Coping Appraisal’. The perceived probability of threat, which consists of variables associated with future flooding and the intensity and severity of flood damage, was positively correlated with structural flood preparedness measures, including raising the plinths of houses, tube wells, and toilets, and a few non-structural actions, including storing dry food at home (*r* = 0.237, *p* < 0.05) and developing an evacuation plan (*r* = 0.334, *p* < 0.01). The above-mentioned correlation results may imply that respondents who foresaw the possibility of flooding with increased intensity and severity in the future were more willing to take structural flood risk reduction measures and also prioritized family safety by developing a flood evacuation plan. 

The perceived severity of future flooding, which included the variable related to damage to households, agricultural crops, the degradation of family health status, and the impact on the overall livelihood of people, was positively correlated with raising the plinths of houses, tube wells, and toilets and a few non-structural actions, such as storing cash money and having emergency equipment and an evacuation plan. The analysis also found negative correlations between the perceived severity of future flooding and each of the following: early warning (*r* = −0.269, *p* < 0.05), developing a community disaster management plan (*r* = −0.281, *p* < 0.01), and damage insurance (*r* = −0.233, *p* < 0.01). These results may mean that respondents who foresaw negative consequences of future floods were more likely to adopt structural interventions and few family-level flood preparedness measures. 

The fear of future flooding was positively correlated with raising the plinths of houses, tube wells, and toilets and negatively correlated with having emergency equipment at home (*r* = −0.218, *p* < 0.05) and connections with NGOs (*r* = −0.258, *p* < 0.05). These results may explain why respondents who showed low to high levels of fear of flooding were more motivated to enact structural improvements and making connections with external organizations where they could seek assistance during crises (Appendix B). 

Moreover, previous flood experience had negative correlations with raising the plinths of houses (*r* = −0.549, *p* < 0.01), tube wells (*r* = −0.591, *p* < 0.01), and toilets (*r* = −0.631, *p* < 0.01) and positive correlations with storing crop seeds (*r* = 0.248, *p* < 0.01), raising family flood awareness (*r* = 0.350, *p* < 0.01), and developing a disaster management plan (*r* = 0.273, *p* < 0.05). These results may mean that respondents with previous flood experience were more willing to take non-structural measures than to enact structural improvement as they may not have seen the value of it due to the changing nature of floods over time. Similarly, the reliance of respondents on any flood risk reduction measure was negatively correlated with structural actions, which was different from the dependency on NGO risk reduction interventions, where the correlation analysis found positive results, as people may have received some monetary support for structural improvement from NGOs.

The distance of the household was positively correlated with structural actions and negatively correlated with storing seeds, having emergency equipment, raising family awareness, and making connections with NGOs. These results could likely mean that the respondents who lived closer to the river were more motivated towards strengthening household-level structures rather than taking non-structural action. On the contrary, the distance of households from the embankment was negatively correlated with raising the plinths of houses (r = −0.548, *p* < 0.01), tube wells (r = −0.425, *p* < 0.01), and toilets (r = −0.443, *p* < 0.01) and with family-level flood awareness (r = 0.334, *p* < 0.01), which may mean that the greater the distance of households from the embankment was, the less willing respondents were to adopt structural measures (Appendix C). 

Further, gender was also seen to have positive correlations with raising the plinth of the house (r = 0.210, *p* < 0.05), having an evacuation plan (r = 0.290, *p* < 0.01), and defining a place for family relocation (r = 0.278, *p* < 0.01) and a negative correlation with the connection with local government. These results may denote that the joint decisions taken by male and female members of a family can influence key structural improvements for family safety. The education and monthly income variables were negatively correlated with the structural improvement of houses, toilets, and tube wells and a few non-structural measures, including storing dry food, crop seeds, and money at home; saving valuable assets; and developing an evacuation plan. These relationships may mean that higher education and monthly income did not necessarily translate into higher motivation towards taking structural flood preparedness measures of households living in flood-prone areas (Appendix C). 

Lastly, the non-responsive attitude showed both positive and negative correlations with the distance of the household from the embankment (*r* = 0.305, *p* < 0.05) and the experience of flooding (*r* = −0.286, *p* < 0.05), respectively (Appendix D). These results may mean that households did not want to take floor preparedness measures where the flood protection embankment was far away and they were less experienced in flooding. 

## 4. Discussion

In alignment with the key components of PMT, this section discusses the research findings along the same directions followed in the previous subsections. 

The overall research findings indicated that the majority of respondents living in the Type 2 and 3 settlements could evaluate ‘Threat’ in line with the perceived probability, severity, and fear of flooding in the future. They foresaw severe flooding happening in the future that could cause serious damage, including damage to household structures and crops. In the Type 1 settlement, the study found positive responses in terms of ‘Threat Appraisal’ from half of respondents, as the rest did not foresee severe flooding coming in the future due to the presence of the flood protection embankment. Those who responded reported having witnessed several embankment breaches in recent years, which caused considerable damage, and they feared flooding of medium to high intensity and severity. As Rahman and Salehin [43] found, the failure of structural flood protection measures can cause higher damage to the economy of areas developed near the embankment than to that of areas away from the embankment. 

Additionally, the correlation analysis showed that respondents who foresaw severe flood threat in the future were more willing and motivated to take structural flood preparedness measures, including the raising of the plinths of houses, toilets, and tube wells, rather than taking non-structural risk reduction measures (also found by [12,18,44]). The study conducted by Mondal et al. [12] also found that a higher proportion of households adopted structural measures, including modifying the house materials with iron sheets. Similarly, Reynaud, Aubert, and Nguyen, in 2013, found that ‘threat appraisal’ gave a significant contribution to elevating the floor of the house and relocating to a safer place in Vietnam [26]. 

Given that structural flood preparedness actions are largely expensive, the present study found that the investment on private structural flood protection action was challenging for people due to limited monthly income (also found by [6]), although many respondents talked about collective action, including collecting mud to raise the plinths of dwellings in the Type 2 and Type 3 settlements. Moreover, those who expected difficulties in earning money after the flood were found to be more interested in taking non-structural protection action that did not require direct financial investment, including storing cash money, crop seeds, and emergency equipment at home and developing an evacuation plan.

The present study also found that the fear of future flooding was positively correlated with the structural flood preparedness measures, which was similar to the findings of the study conducted in Vietnam [26], where the authors explained that the fear of dam collapse might have been a reason for farmers to implement self-preparedness strategies. However, the study conducted in Germany [17] did not find a significant relationship between the fear of future flooding and preparedness action taken by people. 

Regarding the coping strategies, this study found that families living in the Type 3 settlement were better prepared for flood disasters than families from the other two settlements; for instance, the majority of respondents reported awareness about what to do during and after floods [45], and they also showed structural preparedness, including having raised the plinths of houses, toilets, and tube wells. Additionally, the DRR interventions implemented by NGOs also contributed towards motivating people to take protection action against floods. The underlying reason might have been the implication of the financial assistance that had been distributed by NGOs for people to take flood protection measures, including the construction of flood-resistant houses, toilets, and tube wells; livelihood improvements; and activities related to raising awareness in flood-vulnerable communities. 

On the contrary, people living away from the embankment (Type 2) showed preparedness on a few variables without having received assistance for flood risk reduction from NGOs or the government. This level of preparedness can be treated as the outcome of having experienced flood disasters by utilizing traditional knowledge to live with flooding (also found by [14]). In the Type 2 and Type 3 settlements, the study found that informal evacuation discussion and planning among families living with flood vulnerability took place just before the event. In contrast, respondents from the Type 1 area were found to be less prepared for flooding, as they had not experienced the event in a long time [45]. 

The correlation results showed that respondents with previous flood and damage experience may have been more willing to take non-structural preparedness measures, which was similar to the results of the study conducted by Diakakis et al. [46]. However, the studies [12,47] found a different status, reporting that having previous flood experience did not necessarily motivate people to take flood preparedness action for the future. 

The present study further showed that joint decisions made by male and female household members influenced taking structural and non-structural measures, which was dissimilar from a study [12] that reported that households with female heads were less likely to take structural flood protection action. Additionally, the educational status of the respondents was not found to influence taking structural flood protection action, which was similar to the research study [12]. There are studies [26] that reported a positive correlation, as residents with higher education took more preparedness measures and stressed the need of knowledge about flood risk management [48]. 

Thus far, a few studies have confirmed that people can understand and learn from a disaster situation that they experienced in the past to prepare for the future; however, some respondents still do not feel that urge [49,50,51]. The present study found a few reasons for this. Firstly, people living in flood-exposed areas lack adequate financial capacity to invest in flood protective initiatives (see also [6,15]). As the monthly income of flood-vulnerable people is insufficient to run household expenditures, investment in flood preparedness is simply not a priority for people. Secondly, people living in flood-exposed areas usually face recurrent flood disasters and do not have the required time to completely recover and be prepared to face flood disasters again. Thus, people suffer severely again and again and cannot improve their economic status. This recurrent disaster exposure may also be one of the reasons why people are unwilling to prepare for flood disasters. Thirdly, there exists the challenge of accessing the various services offered by the government and nongovernmental sectors; the service channels at the local level are complicated and bureaucratic. For instance, Union Parishad cannot make many decisions without informing higher-level (Upazila and District) offices due to the highly centralized decision-making culture in Bangladesh. Additionally, people need to have location-specific information about flood disasters, including flood intensity and severity, continuous status updates, estimation of damage, etc. Fourthly, the changing pattern of flooding due to the changing climate makes it harder for a vulnerable community to be prepared for flood disasters. For instance, the flood velocity during the 2017 flood and the height of water were much higher than the previous year.

## 5. Conclusions

The study found improved protection motivation status, reflecting threat and coping appraisal, among the respondents living in the Type 3 settlement, as compared with those from the Type 2 and Type 1 settlements. Further, the efficacy of the risk evaluation of future flooding of the respondents living in the Type 2 and Type 3 settlements was found to be higher than that of people living by the embankment (Type 1) in Tangail District in Bangladesh. 

In terms of coping appraisal, the respondents living in the Type 3 area were found to be more motivated towards taking both structural and non-structural measures, including awareness of early flood warning, as they had achieved a better understanding about what to do before, during, and after a flood and had undertaken household-level structural improvement and mitigation measures. In contrast, people living by the embankment (Type 1) were found to be less motivated towards preparing for flooding due to their high reliance on the flood protection embankment. However, around half of respondents was found to be concerned about the waterlogging that happened due to embankment breaches in 2017. The households located away from the embankment (Type 2) also showed motivation towards a few flood preparedness measures. As these people had not been involved in any external risk reduction intervention before, this level of preparedness motivation can be treated as the outcome of having experienced flood disasters utilizing traditional knowledge to live with flooding. Additionally, a few respondents from all areas were found not to be motivated to prepare for flood as they thought it is a natural event and they could not do anything about it. 

In the correlation analysis, threat appraisal (i.e., perceived probability of threats, perceived severity of threat, and fear) was found to have a positive correlation with structural flood risk reduction measures, including raising the plinths of houses, toilets, and tube wells, and non-structural actions, including developing family evacuation and relocation plans with awareness of flooding. Previous flood experience of respondents was not found to influence their motivation to take structural flood risk reduction measures, but it influenced storing crop seeds, mobilizing emergency equipment and increasing family awareness. The reliance on flood risk reduction measures had a negative relation with structural improvements; however, the relationship status changed to positive with NGO interventions that allocated financial assistance to vulnerable families to make the household structures resilient. Socio-economic variables, including education and income, did not have a significant influence on structural protection measures; however, the study found that gender status, with joint decision making at the family level, influenced raising the plinth of the house.

Moreover, the application of PMT in the disaster domain needs to be further understood considering different contexts; hence, it requires more research work to be performed in order to capture the various dimensions of the protection motivation of people living with disaster risk. The existing literature on PMT in the disaster domain has been mostly conducted by employing quantitative methods; however, using mixed methods can provide more comprehensive information along with a qualitative reasoning of people’s motivation to take preparedness measures against flooding. 

Finally, the authors acknowledge that this research study was subject to certain limitations. Firstly, this study was largely built on a quantitative research approach, which correspondingly uncovered a huge scope of future research in terms of deriving qualitative evidence to corroborate the research findings. Furthermore, this study was mainly based on three specific settlements in Bangladesh, due to which further explorations across different geospatial settings and in the context of other disasters are imperative to determine the wider applicability of the derived findings. In the data analysis, the authors could only perform correlation; however, an in-depth statistical analysis would be needed to explore how these variables interact with each other. While the field surveys in this study were mainly conducted in the aftermath of the 2017 flood disaster, the future scope of this research study could also entail the assessment of the implications of COVID-19 on various flood risk reduction measures and the protection motivation status of people living in flood-prone areas. 

## Figures and Tables

**Table 1:** Indicators associated with key PMT components that are studied in this research study (adopted from [17,18,26]).

PMT Component	Key Indicators Studied
General overview of flood risk exposure of the area	Distance of household (HH) from a nearby river considering the flood impact that might happen within a certain time; Distance of HH from flood protection embankment considering the accessibility of households to temporary relocation.
Threat appraisal (hypothetical flooding)	Perceived probability of flooding: hypothetical/future flooding, inundation of household (HH), intensity of flooding, severity of flooding; Perceived severity of flooding: damage to HHs and agricultural crops, death of domestic animals, family health, income struggle, livelihood impacts; Fear of flooding.
Coping appraisal (flood preparedness)	Existence of hard/structural flood protection measures (raising the plinths/basement of houses, tube wells, and toilets); Soft measures (early flood warning, storing dry food, crop seeds, family-level awareness of flood disaster, developing an evacuation and relocation plan, saving valuable assets); Institutional network (having connection with different stakeholders, including GOs and NGOs, for assistance during and after the crisis, including taking loans); Non-responsiveness towards flood protection actions.
Threat experience appraisal	Previous flood experience and associated damage scenario; Access to assistance from stakeholders.
Reliance/belief on DRR intervention	Dependency on flood protection embankment; Dependency on the flood risk reduction interventions of NGOs.
Socio-economic information	Gender; Educational status; Monthly income; Alternative source of income.

**Table 2:** The respondents’ (%) evaluation of intensity and severity, and fear of future flooding in all three settlements, based on a five-point Likert scale ranging from ‘Very Low to Very High’. Source: survey.

Likert Scale of Responses (%)	Type 1 Settlement		Type 2 Settlement		Type 3 Settlement	
	Intensity and Severity of Flooding (n = 15)	Fear of Flooding (n = 30)	Intensity and Severity of Flooding (n = 30)	Fear of Flooding (n = 30)	Intensity and Severity of Flooding (n = 30)	Fear of Flooding (n = 30)
Very High	0	17	14	23	60	73
High	20	47	63	37	17	23
Medium	27	33	3	17	20	0
Low	47	0	7	17	3	0
Very Low	6	0	13	6	0	3

**Table 3:** Why the respondents did not want to take flood preparedness measures in the study area. Source: survey.

Type of Response	Type 1 (n = 17)	Type 2 (n = 11)	Type 3 (n = 5)
Natural event, so I cannot do anything (%)	59	82	80
It would not harm me (%)	29	9	0
I shall get support from others (%)	0	9	20
I am not interested (%)	12	0	0

## Data Availability

Not applicable.

## Appendix A. Survey Questionnaire

The survey questionnaire consisted of a few sections, and questions were developed based on the PMT components mentioned in Table 1.

The first section regarded a general overview on the flood exposure of the respondents, addressing the distance of the household from a nearby river and flood protection embankment, and the possibility of inundation of the house with flood water. The second part was about threat experience appraisal. Binary and multiple-choice questions focusing on previous flood experience of the respondents were added to capture the damage information related to household structures, agriculture crops, family health, etc. The follow-up question was about the status of receiving assistance from the government and nongovernmental organizations during and after the flood in the area.

The third part was about threat appraisal considering hypothetical flood disasters in the future. Here, the respondents were asked about the possible intensity of flooding and severity of damage to houses, crops, fisheries, domestic animals, family health, and the livelihood of affected people caused by future flooding. This section also integrated questions on the level of fear of flooding of respondents using a Likert scale ranging from a very high level of fear to a very low level of fear.

The fourth section was about coping appraisal to assess the short- and long-term flood preparedness measures taken by respondents. These questions were again grouped into three clusters: structural measures (raising the plinths of houses, tube wells, and toilets), non-structural measures (increasing awareness and developing evacuation and disaster management plans), and institutional network (connection with various stakeholders). This section further integrated the reasons behind the non-responsive behavior to flood risk of the respondents and the associated reasoning behind not taking any flood protection action.

The fifth part represented the reliance/belief of the respondents on flood risk reduction measures that could reduce damage due to hypothetical flooding. The flood protection embankment and the disaster risk reduction/resilience project implemented by nongovernmental organizations were considered in this section.

The last part was about socio-economic information of the respondents covering gender, education, monthly income, and sources of income.

## Appendix B

**Table A1:** Correlations among variables of threat appraisal and coping appraisal.

Variables of Threat Appraisal	Coping Appraisal/Flood Preparedness Actions																
	Early Flood Warning	Storing Dry Food	Raising House Plinth	Raising Tube-Well Plinth	Raising Toilet Plinth	Storing Crop Seed	Storing Money	Storing Emergency Equipment	Family Awareness	Evacuation Plan	Saving Valuable Asset	Family Relocation Place	Connection with NGOs	Possibility to Take Loan	Damage Insurance	Community DM Plan	Connection with Local Government
Threat Appraisal: Perceived Probability of Flooding																	
Future flooding	−0.054	**0.237 ***	**0.321 ****	**0.377 ****	**0.377 ****	−0.071	0.064	−0.101	−0.153	**0.334 ****	0.170	0.145	−0.098	0.101	−0.022	0.083	−0.133
Inundation of HH	−0.079	0.019	−0.019	−0.085	−0.029	0.028	−0.105	−0.104	**0.222 ***	0.122	−0.054	0.114	−0.021	−0.031	0.092	**−0.240 ***	−0.070
Intensity of flooding	−0.147	0.095	**0.275 ****	**0.331 ****	**0.331 ****	−0.058	0.071	−0.068	0.081	**0.347 ****	0.005	0.134	0.086	**0.358 ****	−0.096	0.032	0.179
Severity of damage	−0.121	0.165	**0.365 ****	**0.421 ****	**0.421 ****	−0.197	0.099	−0.145	−0.064	**0.302 ****	0.022	0.084	−0.022	0.252 *	−0.110	0.196	0.041
Threat Appraisal: Perceived Severity of Flooding																	
Household damage	**−0.269 ***	−0.128	−0.055	−0.047	−0.047	0.055	−0.158	**−0.252 ***	−0.050	−0.172	−0.142	−0.072	−0.168	**−0.239 ***	−0.016	**−0.281 ****	−0.002
Agricultural-crop damage	0.159	0.082	−0.083	0.003	−0.057	0.138	**0.218 ***	**0.222 ***	**0.226 ***	0.115	−0.063	**0.239 ***	0.023	0.069	0.025	0.034	0.041
Death of domestic animals	0.077	0.185	0.111	**0.221 ***	**0.255 ***	−0.137	−0.096	−0.092	0.045	**0.247 ***	0.125	0.010	0.012	0.252 *	−0.047	0.174	−0.206
Family health status	−0.120	−0.172	**0.214 ***	**0.333****	**0.333****	**−0.269 ***	**−0.239 ***	**−0.214 ***	**−0.265 ***	0.057	−0.084	0.108	−0.164	−0.111	−0.090	−0.025	−0.052
Income struggle	0.093	**0.218 ***	−0.152	−0.119	−0.119	**0.215 ***	**0.353 ****	**0.325 ****	**0.254***	**0.242 ***	0.145	**0.218 ***	0.185	**0.313 ****	0.167	0.056	**0.251 ***
Livelihood impact	−0.119	0.008	**0.350 ****	**0.268 ***	**0.223 ***	−0.061	−0.104	−0.145	−0.012	0.176	0.037	0.078	0.052	0.054	**−0.233 ***	0.012	0.094
Threat Appraisal: Fear of Flooding																	
Fear of flooding	−0.108	−0.009	0.067	**0.325 ****	**0.302 ****	−0.132	0.170	**−0.218 ***	−0.057	0.183	−0.103	−0.159	**−0.258 ***	0.009	−0.026	**0.217 ***	−0.053

** Correlation was significant at the 0.01 level (2-tailed). * Correlation was significant at the 0.05 level (2-tailed).

## Appendix C

**Table A2:** Correlations among variables related to flood experience, reliance, risk exposure, socio-economic status, and coping-appraisal variables.

Variables	Coping Appraisal/Flood Preparedness Actions																
	Early Flood Warning	Storing Dry Food	Raising House Plinth	Raising Tube-Well Plinth	Raising Toilet Plinth	Storing Crop Seed	Storing Money	Emergency Equipment	Family Awareness	Evacuation Plan	Saving Valuable Asset	Family Relocation Place	Connection with NGOs	Possibility to Take Loan	Damage Insurance	Community DM Plan	Connection with Local Government
Threat Experience Appraisal																	
Experienced flooding in the past	0.165	−0.156	**−0.549 ****	**−0.591 ****	**−0.631 ****	**0.284 ****	0.006	**0.276 ****	**0.350 ****	**−0.276 ****	−0.141	−0.062	0.064	0.066	−0.152	**0.273 ***	−0.188
Reliance on DRR Measures																	
Existence of risk reduction measures	**0.276 ****	0.075	**−0.461 ****	**−0.305 ****	**−0.305 ****	0.031	**0.239 ***	0.118	**0.287 ****	−0.043	−0.042	−0.004	−0.106	0.140	0.031	0.194	0.080
Flood embankment	**−0.225 ***	−0.092	−0.150	−0.094	−0.094	0.146	−0.184	0.019	−0.176	**−0.425 ****	−0.040	−0.110	0.027	**−0.338 ****	**−0.333 ****	−0.092	−0.190
NGO’s resilience program	0.116	**0.208 ***	**0.245 ***	**0.298 ****	**0.220 ***	**0.245 ***	0.083	−0.010	0.079	**0.232 ***	**0.340 ****	0.095	0.100	−0.066	0.055	**−0.217 ***	0.134
Risk Exposure																	
Distance of HH from river	−0.047	0.155	**0.474 ****	**0.435 ****	**0.462 ****	**−0.215 ***	0.093	**−0.219 ***	**−0.305 ****	0.179	0.176	0.015	**−0.251 ***	−0.102	−0.002	0.087	0.173
Distance of HH from embankment	0.172	−0.035	**−0.548 ****	**−0.425 ****	**−0.443 ****	0.158	**0.245 ***	0.112	**0.334 ****	−0.172	−0.184	0.057	−0.012	−0.035	−0.035	0.136	−0.184
**Socio−Economic Status**																	
Gender	0.093	−0.071	**0.210 ***	0.082	0.082	0.148	0.086	0.115	0.033	**0.290 ****	0.183	**0.278 ****	0.098	−0.086	0.182	0.049	**−0.231 ***
Educational status	−0.075	**−0.300 ****	**−0.219 ***	**−0.270 ****	**−0.232 ***	**−0.234 ***	**−0.298 ****	−0.137	−0.167	**−0.273 ****	**−0.394 ****	−0.198	0.007	−0.056	−0.090	−0.075	−0.086
Monthly income	0.150	**−0.387 ****	**−0.385 ****	**−0.464 ****	**−0.522 ****	0.086	0.053	0.053	0.101	**−0.252 ***	−0.085	−0.098	−0.046	0.092	−0.132	0.163	−0.174

** Correlation was significant at the 0.01 level (2-tailed). * Correlation was significant at the 0.05 level (2-tailed).

## Appendix D

**Table A3:** Correlations between non-responsive attitude and socio-economic and risk-exposure variables.

Variable	Gender	Educational Status	Monthly Income	Distance of HH from River	Distance of HH from Embankment	Intensity of Future Flooding	Severity of Damage	Existing Risk Reduction Measures	Level of Fear Considering Flooding	Experienced Flooding in the Past
Non-responsive attitude	0.102	−0.084	−0.184	**0.305** *****	−0.269	0.171	0.202	−0.189	0.099	**−0.286** *****

* Correlation is significant at the 0.05 level (2-tailed).
